# Effectiveness of Solvent Vapor Annealing over Thermal Annealing on the Photovoltaic Performance of Non-Fullerene Acceptor Based BHJ Solar Cells

**DOI:** 10.1038/s41598-019-44232-0

**Published:** 2019-06-12

**Authors:** Ram Datt, Suman, A. Bagui, Afzal Siddiqui, R. Sharma, Vinay Gupta, S. Yoo, S. Kumar, Surya Prakash Singh

**Affiliations:** 1 CSIR-National Physical Laboratory, Dr. K. S. Krishnan Marg, New Delhi, 110012 India; 2 Polymers and Functional Materials Division, CSIR-Indian Institute of Chemical Technology (IICT), Uppal road, Tarnaka, Hyderabad, 500007 India; 3 Academy of Scientific and Innovative Research (AcSIR), Ghaziabad, 201002 India; 4 Department of Electrical Engineering, Korea Advanced Institute of Science and Technology (KAIST), 291 Daehak-ro, Yuseong-gu, Daejeon Republic of Korea; 5 Department of Mechanical and Materials Engineering, Khalifa University of Science and Technology, Masdar Institute, Masdar City, P.O. Box 54224 Abu Dhabi UAE

**Keywords:** Energy, Devices for energy harvesting

## Abstract

We explore two small molecules containing arms of dicyano-n-hexylrhodanine and diathiafulvalene wings terminated with benzothiadiazole linker, denoted as BAF-4CN and BAF-2HDT, respectively, as small molecule non-fullerene acceptors (SMNFAs) in organic solar cells. The proposed materials are mixed with a low band gap polymer donor PTB7-Th having broad absorption in the range of 400–750 nm to form solution-processed bulk heterojunctions (BHJs). The photoluminescence (PL) measurements show that both donor and acceptor can quench each other’s PL effectively, implying that not only electrons are transferred from PTB7-Th → SMNFAs but also holes are transferred from SMNFAs → PTB7-Th for efficient photocurrent generation. Furthermore, solvent vapor annealing (SVA) processing is shown to yield a more balanced hole and electron mobility and thus suppresses the trap-assisted recombination significantly. With this dual charge transfer enabled via fine-tuning of end-groups and SVA treatment, power conversion efficiency of approximately 10% is achieved, demonstrating the feasibility of the proposed approach.

## Introduction

The organic photovoltaic (OPV) devices based on bulk hetero-junction (BHJ) active layer, which is a blend of electron donor (D) and acceptor (A) components in the bulk, have attracted enormous attention for solar cell applications due to their simple solution process techniques such as spin coating, spraying, stamping, printing, and doctor blading. The OPV devices have advantages of light-weight, facile roll-to-roll production, low-cost, efficient exciton dissociation (*η*_*ED*_ ~1) and ease of device engineering^1,2^. In order to improve the photovoltaic performance of solution processed BHJ solar cells, various types of donor-acceptor combination including polymer donor (PD)/polymer acceptor (PA)^3^, small molecular donor (SMD)/small molecular acceptor (SMA)^4,5^, PD/SMA^6,7^, and SMD/PA^8^ have been endeavored.However, solution processable small molecules have certain advantages over their conjugated polymer counterparts as the former have well-defined molecular structures, definite molecular weight, a higher degree of purity and absence of batch-to-batch variation during production^9,10^. In the case of SMAs, most of the research groups are mainly modifying fullerene (C_60_) and its derivatives – phenyl-C_61_-butyric acid methyl ester (PC_61_BM) and phenyl-C_71_-butyric acid methyl ester (PC_71_BM) only. In the past few years, fullerene derivatives have shown promising power conversion efficiencies (PCEs) over 10% in BHJ solar cells with various polymer and small molecule donors^11^. However, fullerene derivatives have some significant drawbacks such as weak absorption in the visible region of the solar spectrum, high production cost, wide band gap, the difficulty of synthesis and purification and low open circuit voltage (V_oc_)^12^.

Recently, small molecule nonfullerene acceptors (SMNFAs) have come up as an alternative to fullerene derivatives and shows considerably high PCEs due to their easy tunability of the electronic and optical properties^13–16^. Significant research efforts have been made to design and synthesize various SMNFAs with different electron-withdrawing groups (EWG) and achieved high PCEs over 14.1% for OSCs^15–18^. The SMNFAs labeled as FBM, CBM and CDTBM bearing dicyano terminated benzothiadiazole unit were synthesized by K. Wanget *et al*. having PCE values of 5.1, 5.3 and 5.0%, respectively when blended with a narrow band gap polymer donor poly[4,8-bis(5-(2-ethylhexyl)thiophen-2-yl)benzo[1,2-b;4,5-b′]-dithiophene-2,6-diyl-alt-(4-(2-ethylhexyl)-3-fluorothieno[3,4-b] thiophene)-2-carboxylate-2,6-diyl)] (PTB7-Th)^19^. Y. Lin group designed and synthesized SMNFAs Indacenodithiophene and Indacenodithieno [3,2-b] thiophene moieties (ladder type electron donor unit) with end cap of dicyanoindane-3-dione named as IEIC and ITIC achieving PCE up to 6.31 and 6.8%, respectively with PTB7-Th donor polymer^20,21^. A number of other ladder type SMNFAs have been recently developed for solution processable BHJ solar cell applications and achieved high PCEs of 12%^22^. To design SMNFAs, proper incorporation of EWGs is key factor, which in turn decreases the lowest unoccupied molecular orbital (LUMO) energy level and promotes the dissociation of excitons. The commonly used EWGs are cyano, TDPP, benzothiadiazole, amide, imide and rhodanine groups. Particularly, the EWGs benzothiadiazole and rhodanine are easily synthesized substituents, which extend light absorption and improve electron mobility of the resultant molecule after attaching them to π-conjugated backbone^5,23–26^.

**Figure 1:**
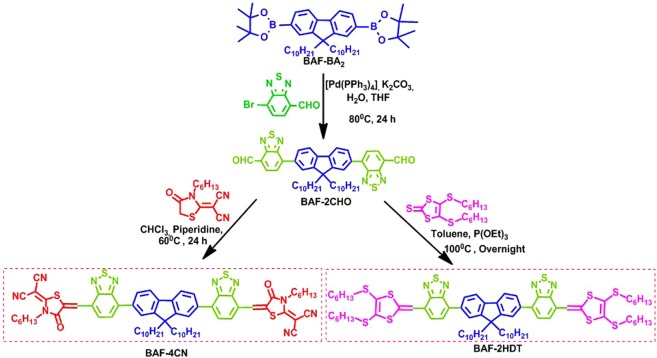
Synthetic route of synthesized non-fullerene acceptors BAF-4CN and BAF-2HDT.

Our group has designed and synthesized two calamitic type SMNFAs coded as BAF-4CN and BAF-2HDT (Fig. 1) having dialkylated fluorene as the core, the electron accepting unit benzothiadiazole (BT) attached to the weak electron donating core fluorene and terminated with dicyano-N-hexylrhodanine (2CN) in the case of BAF-4CN and diathiafulvalene (HDT) in the case of BAF-2HDT for use in solution processable OPV cells^27,28^. Introduction of dicyano-n-hexylrhodanine and HDT substituent as end-capping agents effectively lowers the LUMO energy level of the resultant molecules, increases the light absorption properties, facilitates delocalization of *π*-electrons, and reduces the band gap and increases the charge transfer mobility by extending linear conjugation throughout the molecular backbone. The solubility and aggregation behavior of these calamitic type SMNFAs have been improved by attaching didecyl chains to fluorene core and hexyl chains to the end caps of the respective molecules. Previously we have reported a highest PCE of 7.1 and 8.4% in solar cells fabricated from these acceptors blending with a low band gap polymer donor poly[(5,6-difluoro-2,1,3-benzothiadiazol-4,7-diyl)-alt-(3,3″′-di(2-octyldodecyl)-2,2′;5′,2″;5″,2″′-quaterthiophen-5,5″′-diyl)] (PffBT4T-2OD). However, fabricating devices from this conjugated polymer is very complex as it requires extra steps such as preheating of ITO coated glass substrates at 110 °C prior to spin-coating of the photoactive layer. PffBT4T-2OD also shows a strong temperature-dependent aggregation behavior in both film and solution which is tedious to control. Moreover, a very high film thickness (~300 nm) of the photoactive layer is required to fabricate devices^29^. These requirements are so stringent that it is not so easy to achieve high PCE from the PffBT4T-2OD based OPV devices and hence there are only a few reports on the PffBT4T-2OD:PC_70_BM based OSCs. In this communication, we have studied the photovoltaic performance of the acceptors in BHJ inverted structure solar cell blending with BDT-based π-electron rich conjugated polymer donor PTB7-Th, commonly known as PCE10 or, PBDTTT-EFT^30,31^. The PTB7-Th based OSCs does not require any sophisticated fabrication condition associated with PffBT4T-2OD. This low band gap (1.58 eV) polymer is thermally stable as well. The BDT unit exhibits a large planar conjugated structure that enhances the charge carrier mobility of the polymer. Functional side groups employed on the central benzene core at 4^th^ and 8^th^ position increase the solubility and maintain the planarity of the BDT unit. The thieno [3,4-b]- thiophene (TT) units are widely used to stabilize the quinoid characteristic of the backbone, leading to the low band gap of resulting polymers. Fluorine atom on TT unit is normally introduced to achieve low-lying highest occupied molecular orbital (HOMO) and LUMO energy levels^32,33^.

## Materials and Methods

### Synthesis and characterization of BAF-4CN AND BAF-2HDT

The synthetic routes of BAF-4CN and BAF-2HDT are depicted in Fig. 1. Intermediate BAF-2CHO was synthesized by Suzuki-cross coupling between BAF-BA_2_ and Br-BT-CHO reactants using [Pd(PPh_3_)_4_] as the catalyst. Knoevenagel condensation between dicyano-N-hexylrhodanine and aryl dialdehyde intermediate (BAF-2CHO) to afford the BAF-4CN in dark red color. Th**e** BAF-2HDT was obtained as dark pink color through Horner–Wittig reaction between intermediate BAF-2CHO and HDT^27,28^. BAF-4CN and BAF-2HDT are readily soluble in common organic solvents, such as dichloromethane (DCM), tetrahydrofuran (THF), chlorobenzene (CB), o-dichlorobenzene (o-DCB) and chloroform at room temperature. ^1^H NMR, ^13^C NMR, and matrix-assisted laser desorption/ionization-time-of-flight mass spectrometry (MALDI-TOF MS) were used to characterize the purity and chemical structures of BAF-4CN and BAF-2HDT.

### Device fabrication

The commercially available ITO-coated glass substrates with sheet resistance approximately 12 Ω/cm^2^ were used as a bottom electrode. First, the substrates were cleaned thoroughly in the soap solution and then ultra-sonicated in de-ionized water, acetone, and isopropyl alcohol sequentially for 15 min in each case. After drying under anhydrous nitrogen flow, the substrates were treated under the flow of ozone gas for 15 min. The zinc oxide (*ZnO*) solution was prepared by sol-gel method and spun onto ITO substrates at 5000 rpm for 1 min to obtain a film thickness of about 40 nm. The *ZnO* electron transport layer was subsequently annealed at 200 °C in the air. The photo-active layer solution was prepared by blending PTB7-Th (1-material) and SMNFAs with a weight ratio of 1:1.5 in chlorobenzene (CB) with PTB7-Th concentration of 10 mg·ml^−1^. The 1,8-Diiodooctane (DIO) 3% and 1-Chloronaphthalene (CN) 3% additives were used. All solutions were stirred overnight on a hotplate set at 80 °C. The warm active layer blend solution was spun on prebaked (at 70 °C) *ZnO* coated ITO substrates at 1000 rpm for 20 s to achieve a thickness of ∼110 ± 10 nm. After that films were either i) thermally annealed at 80 °C for 15 min, ii) transferred to glove box ante-chamber immediately after spin coating for slow vacuum annealing at room temperature for 2 h, or, iii) separately put into a petri-dish with few drops of CB for solvent vapor annealing for 30 min^34^. Then the substrates were transferred to a thermal evaporation chamber at a base pressure below 5 × 10^−6^ mbar. A 10 nm thick hole transport layer of molybdenum oxide (*MoO*_3_), purchased from Alfa Aesar (99.9995% pure), was first deposited through a proper shadow mask and finally, 100 nm thick silver (*Ag*) (Alfa Aesar, 99.999% pure) was deposited to form the top electrode.

### Device characterization

Photovoltaic characterization of the fabricated devices was done using a Keithley 2600 source meter and a CEP-25ML Spectral Response Measurement System under simulated AM 1.5 G irradiance of 100 mW·cm^−2^ intensity. The solar simulator was calibrated by an NREL-certified reference cell before the measurements. The EQE spectra of the solar cells were measured using a standard lock-in amplifier and monochromator. The devices were encapsulated inside a *N*_2_ filled glove box using UV-epoxy before any electrical measurement. All the measurements have been carried out with 10 mm^2^ active area.

**Figure 2:**
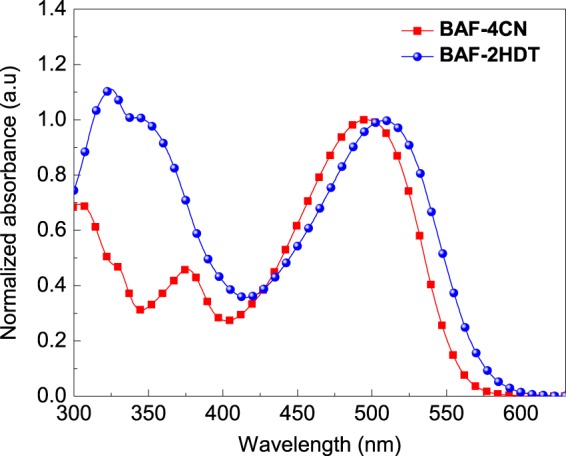
UV-vis absorption spectra of BAF-4CN and BAF-2HDT in chloroform solution.

Figure 2 shows the UV-visible optical absorption spectra of both the SMNFAs. The molecules BAF-4CN and BAF-2HDT solution exhibited strong and broad absorption in the wavelength range between 300–580 nm, originated from π-π* transition and intra-molecular charge transfer (ICT). The HOMO/LUMO energy levels of BAF-4CN and BAF-2HDT were found to be −5.71/3.61 eV and −5.69/3.58 eV, respectively. The values of optical band gaps (*E*_*0-0*_) of BAF-4CN and BAF-2HDT were obtained from the tangent of the absorption spectra in chloroform solution and are found to be 2.16 eV and 2.11 eV, respectively.

**Figure 3:**
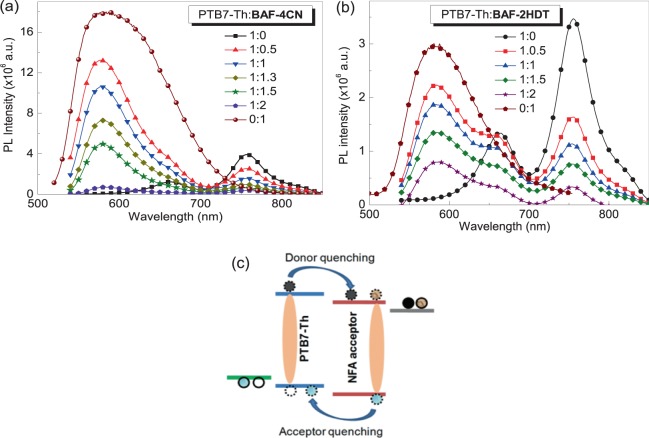
Photoluminescence quenching of PTB7-Th in the presence of (**a**) BAF-4CN and (**b**) BAF-2HDT, (**c**) schematic representation of the ‘electron-hole transfer’ model for NFAs.

The photoluminescence quenching behavior of BAF-4CN and BAF-2HDT were investigated in bulk-composite films and results are illustrated in the Fig. 3a,b, respectively. A gradual quenching of PTB7-Th peak at 758 nm was observed by a monotonic increment of the acceptor content in both blend films when excited at 480 nm. This indicates a highly efficient photoinduced charge transfer from donor to the acceptor molecules.

The photoluminescence quenching behavior in BAF-4CN and BAF-2HDT based BHJ film is illustrated in the Fig. 3a,b, respectively. There is a strong quenching of PTB7-Th by both BAF-4CN or BAF-2HDT which indicates its good electron acceptor characteristics. However a novel feature in the Fig. 3a,b is that PTB7-Th also quenches both BAF-4CN or BAF-2HDT^35,36^. This phenomenon is not observed in the case of fullerene based acceptors; possibly due to the fact the absorption of fullerene acceptors is quite low whereas in the case of NFAs, strong light absorption is observed. This would leads to exciton creations in the NFAs and dissociation at the interface, leading to a hole transfer to the donor. This is illustrated in Fig. 3c, where an electron is transferred from donor the NFA, whereas a hole is transferred from NFA to the donor. Therefore in this case, both donor and acceptor are indistinguishable therefore such NFAs should be denoted as n-type.

**Figure 4:**
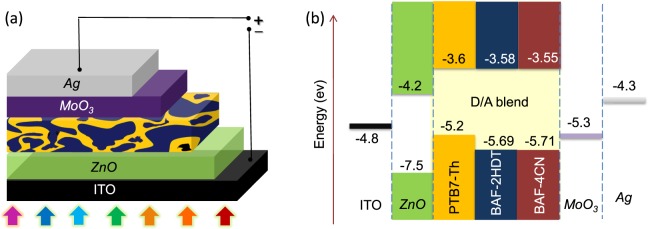
(**a**) Schematic representation of the device structure – ITO/*ZnO*/photoactive layer/*MoO*_3_/*Ag*; (**b**) corresponding energy band diagram of the device (not in scale). All the energy level values are in eV unit.

**Figure 5:**
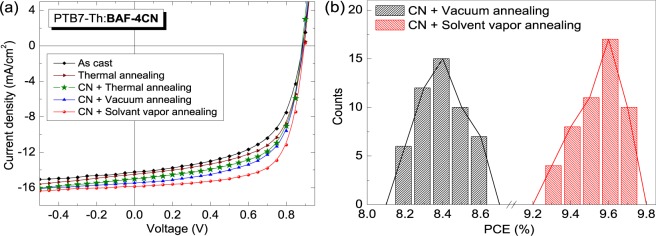
(**a**) Characteristic *J-V* curves of PTB7-Th:BAF-4CN based OPV devices under various fabrication conditions; (**b**) the efficiency histograms for the best performing solar cell.

**Figure 6:**
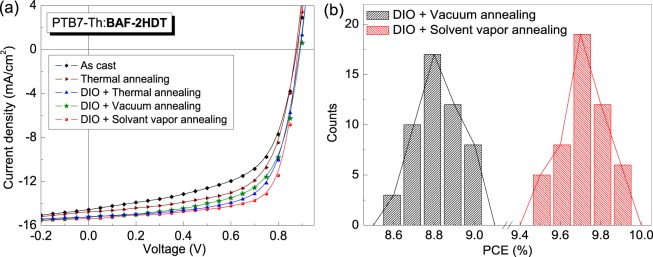
(**a**) The *J-V* characteristics of PTB7-Th:BAF-2HDT based OPV devices constructed under various fabrication conditions; (**b**) the efficiency histograms for the best performing solar cell.

The device structure and energy band diagrams of two SMNFAs BHJ solar cell devices blended with low band gap polymer PTB7-Th are schematically illustrated in Fig. 4a,b. Thin film BHJ solar cells in inverted device structure ITO/*ZnO*/PTB7-Th:SMNFAs/*MoO*_3_/*Ag* were fabricated and tested under simulated solar illumination of intensity 100 mW∙cm^−2^, *i*.*e*.1 sun. The BHJ solar cells with optimized donor-to-acceptor (D/A) weight ratio were cast from chlorobenzene with an active layer thickness of 110 ± 10 nm. The current density-voltage (*J-V*) characteristics of PTB7-Th:BAF-4CN and PTB7-Th:BAF-2HDT based devices under different experimental conditions are shown in Figs 5 and 6, respectively and the photovoltaic parameters of the devices are summarized in Table 1.

**Table 1:** Summary of photovoltaic parameters of BHJ solar cells constructed from PTB7-Th:BAF-4CN and PTB7-Th:BAF-2HDT blends

SMNFAs	Additives 3 vol/vol %	Treatments	*V*_*oc*_ (V)	*J*_*sc*_ (mA/cm^2^)	*FF* (*%*)	*PCE* (%) (Av.)
BAF-4CN	—	As cast	0.889	14.2	57.7	7.3 (7.1)^a^
	—	TA	0.886	14.5	59.0	7.6 (7.3)^a^
	DIO	TA	0.884	14.9	60.2	7.9 (7.7)^a^
	CN	TA	0.885	15.0	61.5	8.2 (7.9)^a^
	CN	VA	0.890	15.4	62.5	8.6 (8.4)^b^
	CN	SVA	0.898	15.8	68.1	9.7 (9.5)^b^
BAF-2HDT	—	As cast	0.878	14.6	59.0	7.5 (7.1)^a^
	—	TA	0.880	14.8	63.5	8.2 (7.9)^a^
	CN	TA	0.885	14.9	63.8	8.4 (8.0)^a^
	DIO	TA	0.896	15.2	64.7	8.8 (8.5)^b^
	DIO	VA	0.892	15.3	66.1	9.0 (8.8)^b^
	DIO	SVA	0.902	15.4	71.5	9.9 (9.7)^b^
PC_71_BM	DIO	SVA	0.800	15.2	69.8	8.5 (8.4)^a^
PC_71_BM	CN	SVA	0.791	12.9	57.5	5.9 (5.7)^a^
FBR	DIO	SVA	0.842	14.3	65.2	7.8 (7.6)^a^
FBR	CN	SVA	0.825	13.7	66.4	7.5 (7.2)^a^

Foot note of table: TA-Thermal annealing, VA-Vacuum annealing, SVA-Solvent vapor annealing. (Average PCE) a = 20 devices, b = 50 devices.

The solar cell made from ‘as cast’ PTB7-Th:BAF-4CN blend films without using any additive or, performing any post-film formation treatment showed a PCE of 7.3% with a *V*_*oc*_ of 0.889 V, *J*_*sc*_ of 14.2 mA∙cm^−2^ and *FF* of 0.58 (Fig. 5a). The PCE of the device is increased marginally to 7.6% upon thermal annealing of the film. Active layer solutions were also prepared using two additives viz. 1,8-diiodooctane (DIO) and 1-chloronaphthalene (CN) to improve solubility of the molecules further. However, the additive CN is found to be more compatible with PTB7-Th:BAF-4CN blend system compared to DIO as can be seen from Table 1. The best performing OPV device fabricated using CN additive (optimized 3 vol/vol %) showed a PCE of 8.1% with a *V*_*oc*_ of 0.884 V, *J*_*sc*_ of 15.0 mA∙cm^−2^ and *FF* of 0.62. In this set of devices, the photo-active layer was thermally annealed (TA) at 80 °C for 15 minutes after spin casting. In order to improve the device performance further, we have investigated the effect of vacuum annealing (VA) and solvent vapor annealing (SVA) treatments on the blend films^27,28^. The VA was carried out inside the antechamber of glove box for the duration of 2 h. For the SVA method, films were separately put into a Petri dish with few drops of CB around it for 30 min. During the different annealing processes, all the films were kept in the glove box and then transferred to the vapor deposition system. The best performing vacuum annealed solar cell showed a PCE of 8.6% with a *V*_*oc*_ of 0.890 V, *J*_*sc*_ of 15.4 mA·cm^−2^ and *FF* of 0.62. On the other hand, the samples which were undergone through SVA showed a maximum PCE of 9.7%, *V*_*oc*_ of 0.898 V, *J*_*sc*_ of 15.8 mA·cm^−2^ and *FF* of 0.68. As can be seen from Table 1, the significant improvements in the value of *J*_*sc*_ and *FF* contributed to this 33% enhancement in PCE compared to the as-cast device.

In the case of the PTB7-Th:BAF-2HDT based BHJ devices, the ‘as cast’ and ‘TA’ (without additive) solar cells showed comparatively higher PCEs of 7.5 and 8.2%, respectively (Fig. 6a). The CN additive marginally improves the PCE from 8.2 to 8.4%. Interestingly, the additive DIO showed better compatibility with BAF-2HDT unlike CN with BAF-4CN. The PTB7-Th:BAF-2HDT based BHJ devices achieved highest PCEs of 8.8, 9.0 and 9.9% fabricated from TA, VA and SVA films, respectively while using DIO (optimized 3 vol/vol %) as the additive. A very high *V*_*oc*_ above 0.9 V and *FF* above 0.7 was observed in the SVA devices. The overall PCE is higher than that ofPTB7-Th:PC_71_BM under the SVA device fabrication condition. Figure 6b shows the efficiency histograms for the best devices where the photo-active layers were treated with VA and SVA treatments. Six cells out of total fifty devices made from SVA treatment showed the highest PCE of 9.9%, whereas nineteen cells showed the average PCE of 9.7%. These results clearly reveal that SVA treatment can be conveniently used to enhance the photocurrent and achieve high PCE in organic solar cells fabricated from SMNFAs^11^.

It is important to note that the LUMO energy levels of SMNFAs are lying very close to that of the donor molecule PTB7-Th, which is at −3.6 eV (Fig. 4b). Conventionally, the energy of acceptor’s LUMO is lower than that of donor’s LUMO significantly and the LUMO-LUMO offset between donor and acceptor is well-known to be the driving force for charge-separation. Absorption of photons leads to the formation of excitons in organic semiconductors. Due to weak electronic intermolecular interaction inorganic materials, the formation of such excitons usually has a high binding energy in the range of hundreds or, sub-hundred of meV. In the conventional case, exciton diffuses towards donor-acceptor (D-A) interface present in BHJ solar cell after its formation. Then exciton relaxes first to a charge transfer (CT) exciton state and involves emission of phonons at the D-A interface site^37^. Excess energy generated through phonons impact back to CT exciton and dissociates it into free charge carriers^38^.

However, in the case of present scenario, where the LUMO energy levels of donor and acceptor are very close to each other, the creation of phonon can’t be the sole governing factor for efficient exciton dissociation. The reason can be understood from the point of a very good absorption spectrum of the active layer over a wider range of wavelength as shown in Fig. 3. It is reported by Grancini *et al*.^39^ that surplus energy with respect to the optical gap is utilized to achieve higher charge generation efficiency. This can be qualitatively understood under the light of different excited states of exciton apart from ground states. Significant excess energy due to photo-excitation causes a large amount of high energy exciton states to be present as well apart from the first excited state. Higher energy states of exciton are more delocalized as compared to the lower lying state because of far less crowding of excitons at higher energy and thus low coupling among themselves^39^. In such case, it is far easier for higher energy hot excitons to dissociate and thus the very low probability of exciton self-recombination. The effect of this phenomenon creates an additional approach for free charge carrier generation which ultimately leads to higher efficiency. Based on aforesaid explanation one can conclude that traditional understanding of exciton dissociation through excess energy by phonon emission is not entirely applicable under the present system of bulk heterojunction, where LUMO-LUMO offset of donor and acceptor is minimal. Instead, excess energy is gained through a good absorption spectrum over a large range creating additional excited states as opposed to conventional understanding.

**Figure 7:**
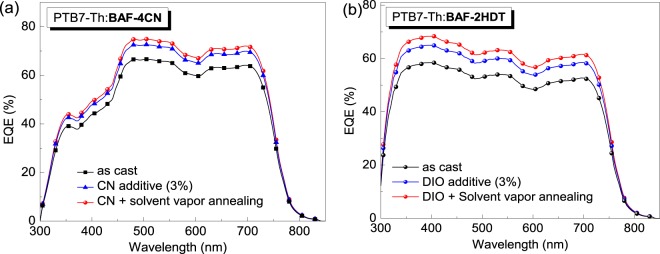
External quantum efficiency (EQE) spectra of (**a**) PTB7-Th:BAF-4CN and (**b**) PTB7-Th:BAF-2HDT based devices under different conditions.

Figure 7a,b show the external quantum efficiency (EQE) profile of PTB7-Th:BAF-4CN and PTB7-Th:BAF-2HDT based OPV devices, respectively fabricated from the films with and without using additives and films treated with SVA. The additive improves the photocurrent and the devices show better EQE compared to the ‘as cast’ OSCs for both the cases. Further, SVA treated devices exhibited best EQE spectra for both the SMNFAs. A maximum EQE value of 74% was observed at 480 nm for BAF-4CN based device (Fig. 7a). On the other hand, BAF-2HDT based OSC showed a maximum EQE of 68% at 398 nm. It can be seen from Fig. 7b that all EQE spectra for PTB7-Th:BAF-2HDT based devices have a broad plateau region in the range of 350 to 750 nm. High values of EQE apprise a very efficient photon-to-electron conversion process in the devices made from both the SMNFAs. It is also worth mentioning that a significant contribution to the spectral response of the solar cells is found in the wavelength range of 480–550 nm for BAF-4CN and 340–480 nm for BAF-2HDT, where the absorption peak of BAF-4CN and BAF-2HDT lies. Thus, it can be concluded that both the donor and acceptors contribute efficiently to EQE and photovoltaic performance of the device.

Insight into the recombination mechanism can be obtained by measuring *V*_oc_ as a function of the light intensity (*I*)^1,2^. At *V*_oc_, the photocurrent is zero, and all photogenerated carriers recombine within the cell. Thus, recombination studies at V_oc_ can provide detailed information about various mechanisms. *V*_oc_ and light intensity (I) are correlated by the following expression,

$$\delta {V}_{oc}=(\frac{kT}{q})\mathrm{ln}(I)+{const}.$$

This implies that the slope of *V*_*oc*_ versus ln(*I*) is equal to *kT*/*q* for bimolecular recombination. In the case of monomolecular Shockley-Read-Hall (SRH) recombination, *n*_e_ and *n*_h_ (at *V*_oc_) would be proportional to the intensity and the slope of *V*_*oc*_ versus ln(*I*) is equal to 2 *kT*/q^40,41^.

**Figure 8:**
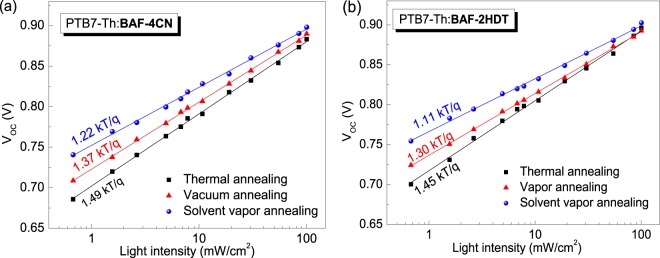
Dependence of *V*_*oc*_ on incident light intensity as observed during light *J-V* characterization of the solar cells made from (**a**) PTB7-Th:BAF-4CN and (**b**) PTB7-Th:BAF-2HDT blends.

**Figure 9:**
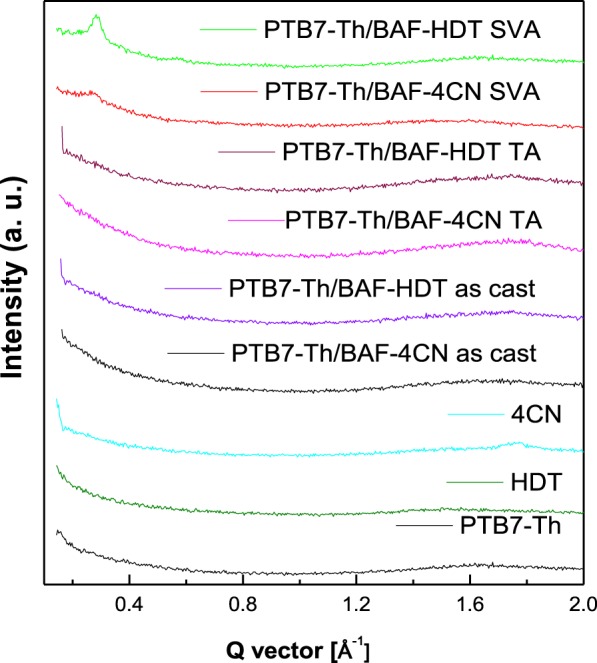
GIXRD graphs of ‘solvent vapor annealed’ PTB7-Th:BAF-4CN and PTB7-Th:BAF-2HDT blend films along with pristine PTB7-Th, BAF-2HDT and BAF-4CN films.

In Fig. 8a,b, the lower value of *KT*/*q* signifies reduced trap-assisted SRH recombination^34,40^, which is lower in the case of SVA BHJ solar cells. Figure 9 shows the grazing incident X-ray diffraction (GIXRD) patterns of pristine PTB7-Th, BAF-2HDT and BAF-4CN films along with their blends. The pure PTB7-Th, BAF-4CN and BAF-2HDT do not show any appreciable peak indicating their amorphous nature. The blend films of PTB7-Th:BAF-4CN and PTB7-Th:BAF-2HDT, as-casted and after thermal annealing also show amorphous nature. However, after SVA treatment XRD peaks are observed at *q* = 0.27 Ả^−1^ and *q* = 0.28 Ả^−1^ for PTB7-Th:BAF-4CN and PTB7-Th:BAF-2HDT, respectively. This peak arises from the (1 0 0) plane of lamellar stacking (out of plain). This shows that SVA reorganize the films better and well organized film morphology is known to result in better PCE. The (1 0 0) coherence length calculated using the Scherrer equation for PTB7-Th:BAF-2HDT blend film is found to be 30 nm in comparison to 20 nm for the PTB7-Th: BAF-4CN blend film. The π∼ π stacking in the in-plane direction at around *q* = 1.7 Ả^−1^ play negligible role^42^.

**Figure 10:**
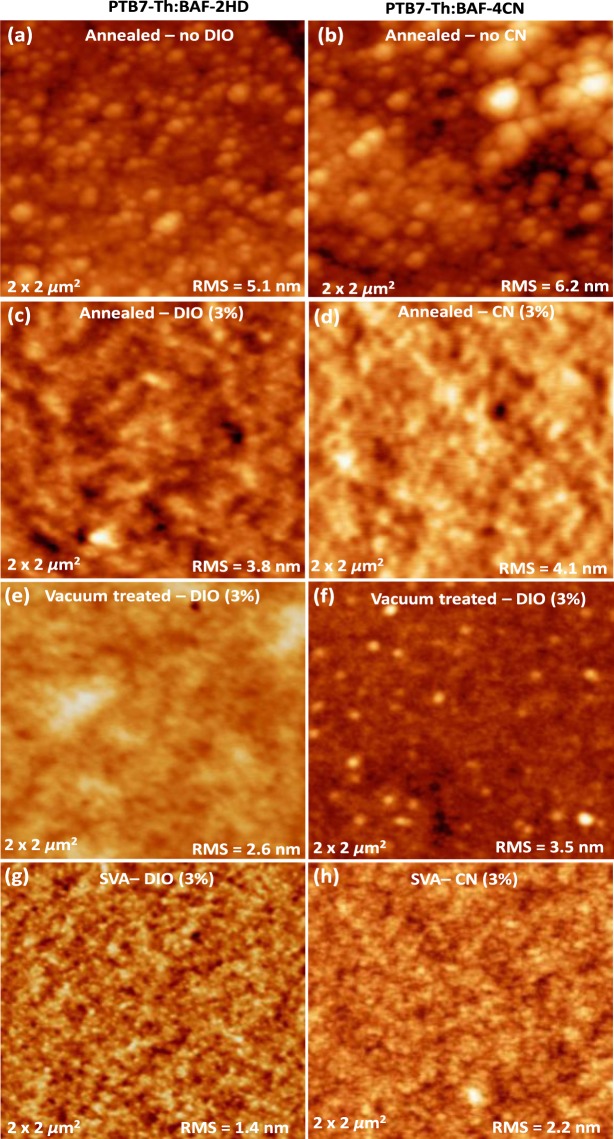
Surface topographic AFM images of (**a**,**b**) annealed without additive, (**c**,**d**) annealed with additive, (**e**,**f**) vacuum treated, and (**g**,**h**) SVA treated blend films for a scanning area of 2 *µ*m × 2 *µ*m. The images on left-hand side represent PTB7-Th:BAF-2HDT blend films, whereas images on right-hand side illustrate PTB7-Th:BAF-4CN bulk composite films.

Surface topology of the PTB7-Th:BAF-2HDT and PTB7-Th:BAF-4CN blend films with various treatments were investigated by atomic force microscopy (AFM) and presented in Fig. 10. The TA PTB7-Th:BAF-2HDT blend films showed a root-mean-square (RMS) roughness 5.1 and 3.8 nm without and with solvent additive (Fig. 10a,c), respectively. A RMS roughness of 2.6 nm was observed for VA blend films (Fig. 10e). However, the RMS roughness significantly decreased to only 1.4 nm with the SVA treatment on the films (Fig. 10g). Very similar trends were also observed in the case of PTB7-Th:BAF-4CN based blend films, where a RMS roughness of 6.2, 4.1, 3.5 and 2.2 nm was observed for ‘TA (without additive)’ (Fig. 10b), ‘TA (with additive)’ (Fig. 10d), ‘VA’ (Fig. 10f) and ‘SVA treated’ (Fig. 10h) active layers, respectively. A smoother morphology of the SVA treated films is consistent with the improved performance of OPVs made thereof.

## Conclusion

We have achieved high efficiency in two fluorene benzothiadiazole based HDT and *n*-hexyl dicyano rhodanine terminated small molecule nonfullerene acceptors using PTB7-Th as a donor for solution processable BHJ solar cells. These two SMNFAs exhibits strong and broad absorption in the range of 300 to 580 nm and appropriate HOMO-LUMO (FMO) energy levels matched with those of PTB7-Th. Both PTB7-Th and NFA shows charge transfer and contribute to the photocurrent. The well**-**balanced hole and electron mobility are achieved in PTB7-Th**:** SMNFAs blends by SVA treatment. BAF-2HDT and BAF-4CN exhibit PCE of 9.9% and 9.7%, respectively, which are the highest values reported so far based on fluorene-based small molecule acceptors.

## References

[1] Facchetti A (2011). π-Conjugated Polymers for Organic Electronics and Photovoltaic Cell Applications. Chemistry of Materials.

[2] Li Y (2012). Molecular Design of Photovoltaic Materials for Polymer Solar Cells: Toward Suitable Electronic Energy Levels and Broad Absorption. Accounts of Chemical Research.

[3] Facchetti A (2013). Polymer donor–polymer acceptor (all-polymer) solar cells. Materials Today.

[4] Liu Y (2013). Solution-processed small-molecule solar cells: breaking the 10% power conversion efficiency. Scientific Reports.

[5] Sharenko A (2013). A High-Performing Solution-Processed Small Molecule:Perylene Diimide Bulk Heterojunction Solar Cell. Advanced Materials.

[6] Nagarjuna P, Bagui A, Hou J, Singh SP (2016). New Electron Acceptor Derived from Fluorene: Synthesis and Its Photovoltaic Properties. The Journal of Physical Chemistry C.

[7] Anthony JE (2011). Small-Molecule, Nonfullerene Acceptors for Polymer Bulk Heterojunction Organic Photovoltaics. Chemistry of Materials.

[8] Cheng P (2014). Towards high-efficiency non-fullerene organic solar cells: Matching small molecule/polymer donor/acceptor. Organic Electronics..

[9] Lin Y, Li Y, Zhan X (2012). Small molecule semiconductors for high-efficiency organic photovoltaics. Chemical Society Reviews.

[10] Walker B, Kim C, Nguyen T-Q (2011). Small Molecule Solution-Processed Bulk Heterojunction Solar Cells. Chemistry of Materials.

[11] Bai H (2015). Nonfullerene acceptors based on extended fused rings flanked with benzothiadiazolylmethylenemalononitrile for polymer solar cells. Journal of Materials Chemistry A.

[12] Anctil A, Babbitt CW, Raffaelle RP, Landi BJ (2011). Material and Energy Intensity of Fullerene Production. Environmental Science & Technology.

[13] Bloking JT (2014). Comparing the Device Physics and Morphology of Polymer Solar Cells Employing Fullerenes and Non-Fullerene Acceptors. *Advanced Energy*. Materials.

[14] Zang Y (2014). Integrated Molecular, Interfacial, and Device Engineering towards High-Performance Non-Fullerene Based Organic Solar Cells. Advanced Materials.

[15] Lin Y (2014). A Star-Shaped Perylene Diimide Electron Acceptor for High-Performance Organic Solar Cells. Advanced Materials.

[16] Yan Q, Zhou Y, Zheng Y-Q, Pei J, Zhao D (2013). Towards rational design of organic electron acceptors for photovoltaics: a study based on perylenediimide derivatives. Chemical Science.

[17] Zhao W (2017). Molecular Optimization Enables over 13% Efficiency in Organic Solar Cells. Journal of the American Chemical Society.

[18] Zhang Y (2018). Nonfullerene Tandem Organic Solar Cells with High Performance of 14.11%. Advanced Materials.

[19] Wang K (2016). π-Bridge-Independent 2-(Benzo[c][1,2,5]thiadiazol-4-ylmethylene)malononitrile-Substituted Nonfullerene Acceptors for Efficient Bulk Heterojunction Solar Cells. Chemistry of Materials.

[20] Lin Y (2015). An Electron Acceptor Challenging Fullerenes for Efficient Polymer Solar Cells. Advanced Materials.

[21] Lin Y (2015). High-performance fullerene-free polymer solar cells with 6.31% efficiency. Energy & Environmental Science.

[22] Li Y (2016). Non-fullerene polymer solar cells based on a selenophene-containing fused-ring acceptor with photovoltaic performance of 8.6%. Energy & Environmental Science.

[23] Lin Y, Li Y, Zhan X (2013). A Solution-Processable Electron Acceptor Based on Dibenzosilole and Diketopyrrolopyrrole for Organic Solar Cells. Advanced Energy Materials.

[24] Guo X, Facchetti A, Marks TJ (2014). Imide- and Amide-Functionalized Polymer Semiconductors. Chemical Reviews.

[25] Wu Y (2015). A planar electron acceptor for efficient polymer solar cells. Energy & Environmental Science.

[26] Li H (2014). Beyond Fullerenes: Design of Nonfullerene Acceptors for Efficient Organic Photovoltaics. Journal of the American Chemical Society.

[27] Suman Bagui A, Gupta V, Maurya KK, Singh SP (2016). High-Performance Non-Fullerene Acceptor Derived from Diathiafulvalene Wings for Solution-Processed Organic Photovoltaics. The Journal of Physical Chemistry C.

[28] Suman Gupta V, Bagui A, Singh SP (2017). Molecular Engineering of Highly Efficient Small Molecule Nonfullerene Acceptor for Organic Solar Cells. Advanced Functional Materials.

[29] Liu Y (2014). Aggregation and morphology control enables multiple cases of high-efficiency polymer solar cells. Nature Communications.

[30] Ye L (2015). Toward efficient non-fullerene polymer solar cells: Selection of donor polymers. Organic Electronics.

[31] Lu Z (2014). Perylene–Diimide Based Non-Fullerene Solar Cells with 4.34% Efficiency through Engineering Surface Donor/Acceptor Compositions. Chemistry of Materials.

[32] Hou J (2009). Synthesis of a Low Band Gap Polymer and Its Application in Highly Efficient Polymer Solar Cells. Journal of the American Chemical Society.

[33] Huo L (2011). Replacing Alkoxy Groups with Alkylthienyl Groups: A Feasible Approach To Improve the Properties of Photovoltaic Polymers. Angewandte Chemie International Edition.

[34] Li Z (2016). High Performance All-Polymer Solar Cells by Synergistic Effects of Fine-Tuned Crystallinity and Solvent Annealing. Journal of the American Chemical Society.

[35] Shivanna Ravichandran, Shoaee Safa, Dimitrov Stoichko, Kandappa Sunil Kumar, Rajaram Sridhar, Durrant James R., Narayan K. S. (2014). Charge generation and transport in efficient organic bulk heterojunction solar cells with a perylene acceptor. Energy Environ. Sci..

[36] Stoltzfus Dani M., Donaghey Jenny E., Armin Ardalan, Shaw Paul E., Burn Paul L., Meredith Paul (2016). Charge Generation Pathways in Organic Solar Cells: Assessing the Contribution from the Electron Acceptor. Chemical Reviews.

[37] Tamura H, Ramon JGS, Bittner ER, Burghardt I (2008). Phonon-Driven Ultrafast Exciton Dissociation at Donor-Acceptor Polymer Heterojunctions. Physical Review Letters.

[38] Scholes, G. D. & Rumbles, G. Excitons in nanoscale systems. *Nature Materials* **5**, 683–696 (2006). 10.1038/nmat1710 16946728

[39] Grancini G., Maiuri M., Fazzi D., Petrozza A., Egelhaaf H-J., Brida D., Cerullo G., Lanzani G. (2012). Hot exciton dissociation in polymer solar cells. Nature Materials.

[40] Gupta V (2013). Barium: An Efficient Cathode Layer for Bulk-heterojunction Solar Cells. Scientific Reports.

[41] Cowan SR, Roy A, Heeger AJ (2010). Recombination in polymer-fullerene bulk heterojunction solar cells. Physical Review B.

[42] Mandoc MM, Veurman W, Koster LJA, de Boer B, Blom PWM (2007). Origin of the Reduced Fill Factor and Photocurrent in MDMO-PPV:PCNEPV All-Polymer Solar Cells. Advanced Functional Materials.

